# Prognostic Factors of Long-Term Outcomes in Endodontic Microsurgery: A Retrospective Cohort Study over Five Years

**DOI:** 10.3390/jcm9072210

**Published:** 2020-07-13

**Authors:** Yeon-Jee Yoo, Dong-Won Kim, Hiran Perinpanayagam, Seung-Ho Baek, Qiang Zhu, Kamran Safavi, Kee-Yeon Kum

**Affiliations:** 1 Department of Comprehensive Treatment Center, Seoul National University Dental Hospital, Seoul 03080, Korea; duswl32@snu.ac.kr; 2 Department of Conservative Dentistry, Dental Research Institute, Seoul National University Dental Hospital, Seoul National University School of Dentistry, Seoul 03080, Korea; tokimdw@naver.com (D.-W.K.); shbaek@snu.ac.kr (S.-H.B.); 3 Schulich School of Medicine & Dentistry, University of Western Ontario, London, ON N6A 5C1, Canada; hperinpa@uwo.ca; 4 Division of Endodontology, Department of Oral Health and Diagnostic Sciences, School of Dental Medicine, University of Connecticut Health Center, Farmington, CT 06030, USA; qzhu@uchc.edu (Q.Z.); safavi@uchc.edu (K.S.); 5 National Dental Care Center for Persons with Special Needs, Seoul National University Dental Hospital for Persons with Special Needs, Seoul 03080, Korea

**Keywords:** cohort study, endodontic microsurgery, long-term outcome, prognostic factors, success, survival rate

## Abstract

The aim of this study was to analyze the long-term outcomes of endodontic microsurgeries in a cohort and identify their association with prognostic factors. A cohort of endodontic microsurgeries followed up periodically with complete clinical and radiographic records for at least 5 years were reviewed retrospectively. Their survival and healing status and profile characteristics were analyzed by Pearson chi-square test and logistic regression (α = 0.05) to identify prognostic factors that influenced outcomes. Of 652 cases in the cohort, 225 (34.5%) were included. The mean follow-up period was 90.4 months (range, 60–168 months). The long-term success rate was 80.5%, and the 5-year survival rate was 83.5%. Logistic regression showed higher success in anteriors compared to molars (OR = 5.405, (95% CI, 1.663–17.571; *p* = 0.005)) and in teeth with crown restorations (OR = 10.232, (95% CI, 3.374–31.024; *p* < 0.001)). Conversely, lower success was found in teeth with periodontal disease (OR = 0.170, (95% CI, 0.032–0.900; *p* = 0.037)) and maxillary sinus involvement (OR = 0.187, (95% CI, 0.035–0.994; *p* = 0.049)). Endodontic microsurgery has a highly favorable long-term outcome. Tooth position, crown restoration, periodontal disease, and maxillary sinus involvement were identified as main prognostic factors.

## 1. Introduction

Endodontic surgery is a treatment of last resort when nonsurgical retreatments fail with persistent lesions from cysts or extraradicular infections [1]. Root-end resections and retrograde fillings eliminate the infection source to promote clinical and radiographic healing as outcome measurements. Success involves resolution of inflammation and regeneration of periodontal ligament and alveolar bone to support normal tooth functions.

Recent advancements in endodontic microsurgery include microscopes, ultrasonic instruments, biocompatible root-end fillings, and miniaturized armamentarium, which have enhanced access, visualization, operative procedures, and tissue responses [2,3]. An early meta-analysis reported that these microsurgeries are 1.58 times more successful than traditional surgeries with cumulative success rates of 93.52% after 6 month follow-up [4]. Their long-term success (>4 years, 82.5%) [5] and survival (8.7 years, 74%) [6] are comparable to nonsurgical retreatment. However, long-term cumulative success rates for microsurgeries in cohorts have not been reported.

To identify the prognostic factors affecting success, retrospective studies have evaluated age, sex, tooth position, obturation length, preoperative lesion size, apical sealing material, and coronal restorations [7,8,9,10,11]. A meta-analysis found that cases without preoperative pain or signs, with dense obturations, with periapical lesions smaller than 5 mm, and operated through microscope were significantly more likely to heal [12]. In a prospective study of 788 surgeries with 4–10 year follow-up, patients over 45 years old, teeth with inadequate obturation length, and crypt sizes smaller than 10 mm had better clinical outcomes [13]. In a 5-year longitudinal study, interproximal bone levels and root-end filling material were significant prognostic factors [14].

Previous studies that relied on short-term follow-up may have overestimated success and failed to account for rates of regression. Longitudinal assessments revealed an 8% reduction in healing rates from 1 (83.8%) to 5 years (75.9%) [14]. Similarly, 6.7% of short-term surgical successes had reverted to failures on long-term follow-up [15]. Additionally, most studies are based on inconsistent surgical databases that include various root-end filling materials, multiple operators, and disparities in surgical devices including microscopes and ultrasonics. Such variations in materials and techniques confound the analysis of prognostic factors.

This study’s purpose was to assess long-term outcomes in cohort endodontic microsurgeries. A cohort of cases performed by a sole practitioner utilizing consistent treatment protocols in teeth that were retained and functional for over 5 years were retrospectively analyzed for healing outcomes to identify the prognostic factors affecting success and survival.

## 2. Materials and Methods

### 2.1. Case Selection

Approval was obtained from the Seoul National University Dental Hospital (SNUDH) Institutional Review Board (ERI19041). Records of patients who underwent endodontic microsurgery performed by a single endodontist between 2006 and 2015 in the Department of Conservative Dentistry at SNUDH and were followed up for at least 5 years were reviewed. Teeth extracted within a 5-year follow-up period were included as failures.

### 2.2. Treatment Protocol

All surgeries were performed by the same endodontist (K.K.) using a microscope (Carl Zeiss, Oberkochen, Germany) and consistent protocols. Following local anesthesia (2% lidocaine, 1:100,000 epinephrine), a full-thickness mucoperiosteal flap was reflected with a P24G periosteal elevator (Hu-Friedy, Chicago, IL, USA). Osteotomy was performed with a #4 round bur to access the root apex, which was resected (≤3 mm) with minimal or no bevel. Granulomatous tissues were curetted and hemostasis obtained with 0.1% epinephrine (Bosmin; Jeil, Seoul, Republic of Korea) and ferric sulfate (Astringedent; Ultradent Products, South Jordan, UT, USA). Root-end surfaces were stained with methylene blue and examined with a micro-mirror to identify cracks, isthmuses, and accessory canals. Apical canal(s) were enlarged deeply (≥3 mm) with ultrasonics (B&L, Ansan, Republic of Korea) and filled with ProRoot MTA (Dentsply Sirona, Tulsa, OK, USA) by micro-pluggers (B&L). Flaps were secured with 5 × 0 coated Vicryl (ETHICON, Bridgewater, NJ and Cincinnati, OH, USA) sutures for 5–7 days and postoperative dressings placed the next day. Antibiotics, analgesics, digestive aids, and 0.2% chlorhexidine gluconate gargle (Hexamedin; Bukwang Phar Co, Ansan, Korea) were prescribed for 5 days.

### 2.3. Clinical and Radiographic Evaluation

**Figure 1:**
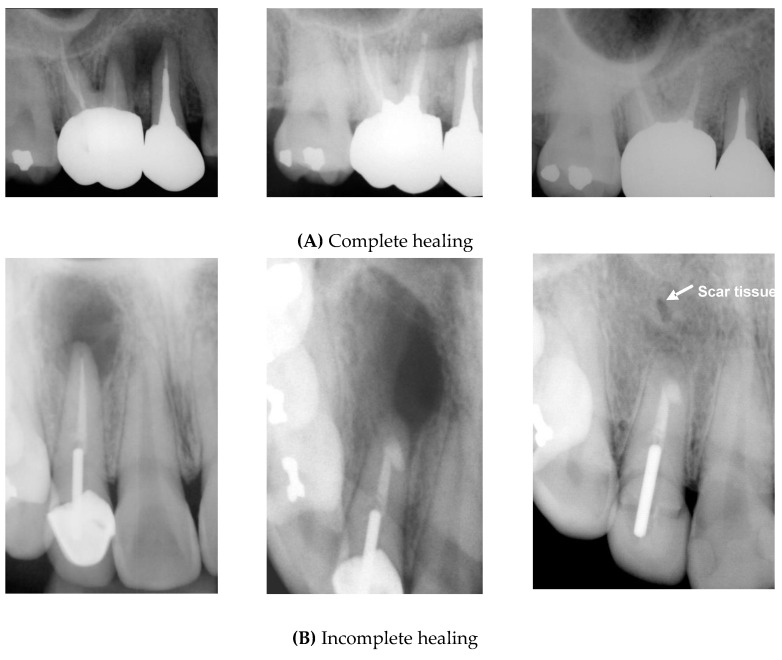

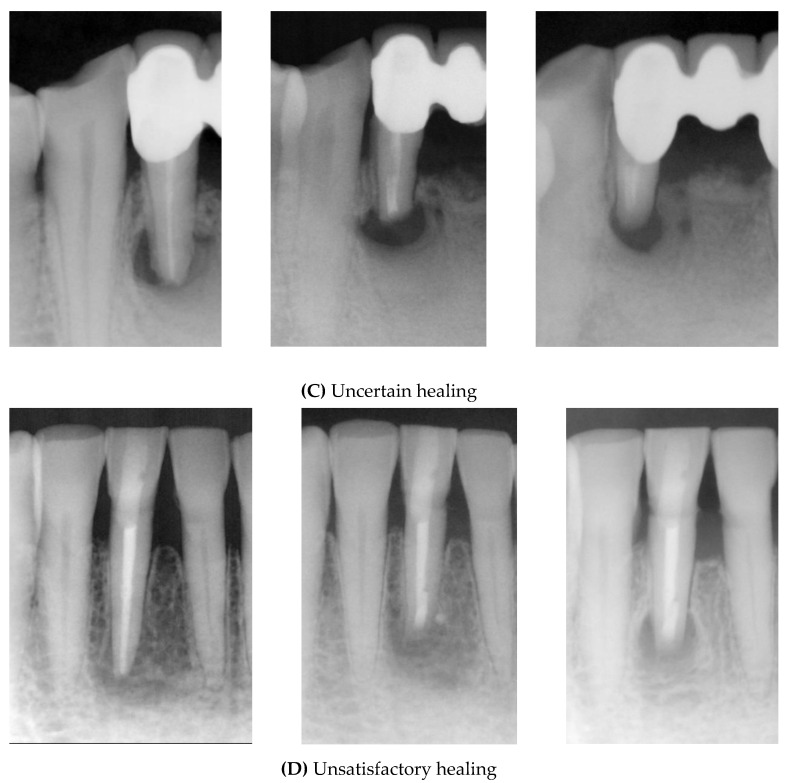
Periapical radiographs demonstrating healing outcomes. (**A**) Complete healing; preoperative (left), postoperative (center), and 14-year follow-up (right) radiographs showing complete healing of #15 and #16. (**B**) Incomplete healing; preoperative (left), postoperative (center), and 14-year follow-up (right) radiographs showing incomplete healing (scar tissue) of #12. (**C**) Uncertain healing; preoperative (left), postoperative (center), and 8-year follow-up (right) radiographs showing uncertain healing of #42. (**D**) Unsatisfactory healing; preoperative (left), postoperative (center), and 4-year follow-up (right) radiographs showing unsatisfactory healing of #41.

Patients were recalled every 6 months for clinical and radiographic examinations. Signs or symptoms of discomfort to palpation and percussion, or biting, tooth mobility, and sinus tracts were recorded. Periapical radiographs were assessed at least annually, and final evaluations based on at least 5-year follow-ups performed independently by two examiners according to Molven’s criteria [16,17]. Each case was assessed as one of the following (Figure 1): 1. Complete healing; 2. Incomplete healing; 3. Uncertain healing; 4. Unsatisfactory healing.

1. Complete healing: the periodontal space has reformed around the apex, which is less than twice the width of the noninvolved parts of the root; complete bone repair with no apical periodontal space.
2. Incomplete healing: the rarefaction has decreased or remained and is characterized by one or more of the following findings: irregular periphery of the rarefaction, the rarefaction is located asymmetrically around the apex, the connection of the rarefaction with the periodontal space is angular, and isolated scar tissue in the bone is observed with these findings.
3. Uncertain healing: rarefaction has decreased in size, and is accompanied by one or more of the following findings: the rarefaction is larger than twice the width of the periodontal space; it has a circular or semicircular periphery; it is located symmetrically around the apex as a funnel-shaped extension of the periodontal space; and bony structures are discernible within the bony cavity.
4. Unsatisfactory healing: the rarefaction has enlarged or has remained unchanged.

Treatment outcomes were based on clinical and radiographic findings. In the absence of clinical signs or symptoms of apical periodontitis, radiographic evidence of complete or incomplete healing was classified as a successful outcome. Conversely, the presence of any clinical signs or symptoms of apical periodontitis and/or radiographic evidence of uncertain or unsatisfactory healing, was classified as a failure. For the computation of survival analysis, teeth that had been extracted within 5 years of follow-up were classified as failures. For each case, 28 preoperative, operative, and postoperative factors were identified [9,18].

1. Preoperative: age, sex, tooth position, jaw, hypertension, diabetes, osteoporosis, length of canal filling, canal filling density, periodontal disease, preoperative pain, percussion, mobility, palpation, bite, swelling, sinus tract, root resorption, lesion size (5 × 5 mm), re-surgery, post
2. Operative: anatomic involvement, bone graft, collagen membrane
3. Postoperative: crown restoration at follow-up, bridge abutment, removable partial denture (RPD) abutment, opposite tooth to implant

### 2.4. Statistical Analysis

Outcomes were assigned to binary variables: success/failure and survival/nonsurvival. All factors were analyzed by Pearson’s chi-square or Fisher’s exact test, and potential prognostic factors were analyzed with a multiple logistic regression. Survival times were calculated from the date of microsurgery to the date of extraction or follow-up confirmation of retention. Cumulative survival rates were calculated by the Kaplan–Meier method. Factors affecting survival rate were evaluated by Cox proportional hazards regression analysis. All statistical analyses were performed with SPSS *v*25.0 software (IBM Corp. Armonk, NY, USA) and α = 0.05.

## 3. Results

Of 652 cases in the cohort, 225 (34.5%) could be recalled to assess outcomes and prognostic factors (Table S1). The mean follow-up was 90.4 months (7.5 years); range of 60–168 months (5–14 years). During surgery, the mean age was 47.8 years, with nearly equal numbers over and under 50 years. Females (162, 72%) outnumbered males (63, 28%). Most were maxillary anteriors (82, 36.4%), followed by maxillary premolars (59, 26.2%), maxillary (31, 13.3%) and mandibular (25, 11.6%) molars, and mandibular anteriors (15, 6.7%) and premolars (13, 5.8%).

For postoperative radiographic interpretation, the inter-rater reliability was high (Cohen’s Kappa coefficient 0.90). There were 148 (66%) completely healed and 33 (14%) incompletely healed cases that accounted for the 80.5% overall success rate (Table 1). There were 37 (16%) unsatisfactory and 7 (4%) uncertain healings. Of 37 unsatisfactory healings, 36 had been extracted within a 5-year follow-up (Table S2) for crown fractures from caries (19%), periodontal disease (19%), or persistent pain (19%).

Chi-square and Fisher’s exact tests for univariate analysis found significant differences in success rates associated with age (*p* = 0.041), tooth position (*p* = 0.023), periodontal disease (*p* = 0.002), tooth mobility (*p* = 0.017), crown restorations (*p* < 0.001), RPD abutments (*p* = 0.007), and sinus involvement (*p* = 0.029) (Table 2).

Subsequently, logistic regression for multivariate analysis found tooth mobility and RPD abutments to be insignificant (*p* > 0.05), so they were excluded to improve the model fitness (R-square). Outcomes were associated with tooth position (OR = 5.405, (95% CI, 1.663–17.571; *p* = 0.005)) and periodontal disease (OR = 0.170, (95% CI, 0.032–0.900; *p* = 0.037)) as preoperative, crown restoration (OR = 10.232, (95% CI, 3.374–31.024; *p* < 0.001)) as postoperative, and anatomic involvement (OR = 0.187, (95% CI, 0.035–0.994; *p* = 0.049)) as operative factors (Table 3).

**Figure 2:**
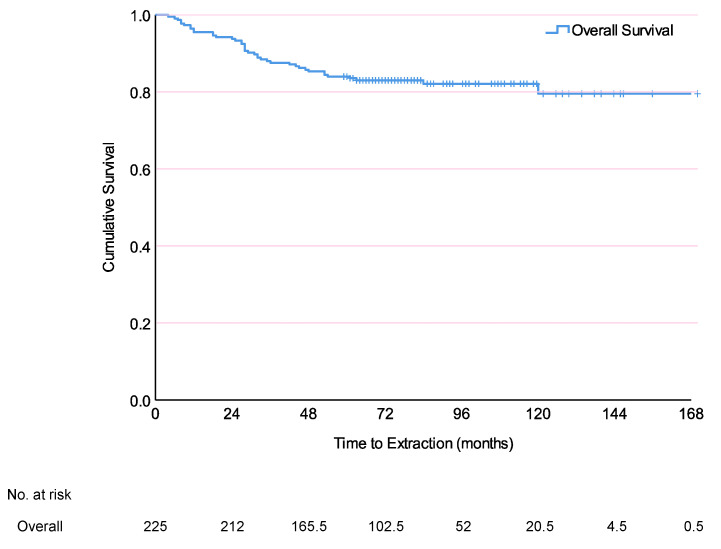
Kaplan–Meier survival analysis of endodontic microsurgery teeth (*n* = 225).

Kaplan–Meier survival analysis showed an 83.5% 5-year survival rate, with mean time until extraction 142.4 months (95% CI, 135.0–150.0) (11.0 ± 0.3 years) regardless of the reason (Figure 2). Cox proportional hazards regression of significant factors from univariate analysis (Table S3) confirmed that survival was significantly affected by tooth position (hazard ratio (HR) = 0.254, (95% CI, 0.098–0.654; *p* = 0.005)) and percussion (HR = 2.078, (95% CI, 1.064–4.058; *p* = 0.032)) among preoperative, crown restorations (HR = 0.166, (95% CI, 0.073–0.376; *p* < 0.001)) and RPD abutments (HR = 8.813, (95% CI, 1.914–40.576; *p* = 0.005)) among postoperative, and sinus involvement (HR = 8.813, (95% CI, 1.378–11.900; *p* = 0.009)) among operative factors (Table S4). Anteriors were more likely to survive, whereas RPD abutments, and teeth with percussion pain or sinus involvement were less likely.

Figure 2 gives the overall survival curve for teeth following endodontic microsurgery. There were 40 extractions in 225 teeth. The 1, 5, and 10-year survival rates were 94.7%, 83.5% and 83.0% respectively. Mean survival time was 142.4 months (135.0–150.0 months).

## 4. Discussion

This cohort’s overall healed rate was 80.5%, which is higher than the Toronto Study’s 74% for 4–10 years [13], or 76% in 5 years longitudinally [14]. This study’s potential success rate was even higher, as some teeth had been extracted for crown fractures from caries (7) and periodontal problems (7) that were unrelated to surgery. Cohort effects aside, higher success may have been due to longer follow-ups. Molven et al. proposed that uncertain cases after 1-year follow-up are unpredictable and need longer recalls [16,17]. Studies have reported that short-term follow-up success rates were 3.5–8.5% lower than for 4-years or longer [14,19]. Additionally, advanced microsurgical tools and techniques including microscopy, ultrasonics, and biocompatible root-end filling materials may have contributed to success. The overall 5-year survival rate was 83.5%, which is similar to that of a smaller recent study [20]. Potential survival rates were even higher if unrelated extractions were excluded.

This retrospective cohort study found that tooth position, crown restorations, periodontal disease, and sinus involvement significantly affected long-term outcomes for endodontic microsurgery. Identifying prognostic factors will enable treatment planning and case selection.

Anterior teeth had the highest success, followed closely by premolars and molars. Differences between anteriors and molars were significant (OR = 5.405), whereas differences between anteriors and premolars were not. Similarly, previous studies showed higher anterior success [9,18,21,22], attributed to ease of surgical access and less complex root canal anatomy than molars [12].

Teeth with adequate coronal restorations at follow-up were ten times more likely to have healed than those without restorations. Furthermore, teeth with full veneer crowns had higher success than those with only composite resins. Similarly, others showed that adequate coronal restorations significantly improved outcomes [8]. Unlike full-crown restorations, composite resins alone may increase endodontically treated teeth’s susceptibility to vertical root fracture [23].

Teeth with combined periodontal-endodontic lesions had significantly less success (OR = 0.170), which is consistent with previous studies [18,24]. Periodontitis causes alveolar bone loss, periodontal recession, apical migration of gingival epithelial cells, and long junctional epithelium, which may compromise healing [10]. They can develop a microbial pathway to the apical region following microsurgery [24]. Therefore, the prognosis for these teeth will depend on both periodontal treatments and endodontic microsurgery [25].

Cases where the periapical lesion had involved the maxillary sinus had much lower success (42.9%). Logistic regression showed that this anatomical involvement significantly reduced the prognosis for microsurgery (OR = 0.187). Lesions involving the sinuses pose microsurgical challenges including difficult access, root-end and granulomatous tissue removal, and the risk of damage and debris into the sinuses [26]. Subsequent healing will likely involve remodeling of the sinus membrane.

Patients under 50 years of age had higher success (85.6%) than those over 50 (74.8%). However, these differences were not significant when logistic regression accounted for other variables. Younger patients may have better healing capacity and less periodontal disease [1], but long-term follow-ups reduced their significance. Previous studies have reported that age and sex did not significantly affect outcomes [9,20,21,27,28].

Interestingly, a history of prior apical surgery on the root did not significantly affect the success of additional microsurgery. Along with other studies [29,30,31,32], this shows that the prognosis is not dependent on prior surgery but on identifying and resolving the cause of apical periodontitis. Teeth fracture resistances (von Mises stress) are not significantly reduced until over 6 mm of root-end resections [33]. Thus, re-surgery can be an effective treatment options for prior inadequate root-end resections and fillings [34].

Systemic conditions including hypertension, diabetes, and osteoporosis did not significantly affect surgical success. Similarly, systemic diseases may not affect nonsurgical endodontics [35]. However, diabetes mellitus can delay periapical healing [36], and diabetics have less success with nonsurgical endodontics, especially for teeth with apical periodontitis [35].

Pre-existing pain and pain on percussion, palpation, or biting were insignificant threats to success, as reported by others [21,22]. However, preoperative mobility diminished success, which was probably due to associated periodontal disease. Similarly, sinus tracts, swellings, root resorption, and lesion size were insignificant. Although others found significant effects for preoperative lesion size [3,37], long-term follow-ups over 5 years allowed sufficient healing in this study. Similarly, 4-year retrospective [8] and 5-year longitudinal studies [14] found that lesion size had an insignificant effect on success. The 4- to 10-year prospective Toronto study found that lesions smaller than 10 mm were more likely to heal [13]. However, lesions over 10 mm may have been incompletely curetted, leaving residual tissues and persistent infections [38].

Serving as fixed partial denture (bridge) abutments did not affect success. However, serving as RPD abutments significantly reduced success according to chi-square tests. Additional logistic regression analysis found the differences to be insignificant, suggesting an uneven distribution of subsets for these variables. Indeed, nonsurgical endodontics was reported to have significantly more failures in RPD abutments than single-crown teeth [39].

Cox proportional hazards regression revealed that tooth position, crown restorations, sinus involvement, RPD abutments, and pain on percussion significantly affected survival. Anterior teeth had a significantly higher cumulative survival than molars (Figure S1A). Full veneer crowns at follow-up significantly increased survival in anteriors and posteriors (Figure S1B–E), despite markedly lower occlusal forces in anterior teeth. Conversely, survival was significantly much lower for cases where the lesion had involved the sinus (Figure S1F). All these results were consistent with the logistic regression analysis of prognostic factors for success.

Pain on percussion was insignificant for success, but significant in Cox proportional hazards regression for survival (Figure S1G). This was likely due to seven extractions from persistent pain, six of which initially had pain on percussion.

RPD abutments had significantly lower survival following surgery (Figure S1H), which is similar to their lower survival following nonsurgical treatment. Additional mechanical stresses imposed on abutments may increase their risk of fracture [40], and the poor crown-root ratio of abutments undergoing microsurgery accounted for lower survival.

To mitigate the effects of confounding variables, a single cohort of consistent surgical cases was studied. However, the residual effects of confounding postoperative factors may have remained. Some failures may not have been due solely to endodontic therapy but to restorative procedures and the periodontal status of the teeth. In this cohort, 34.5% were recalled and included in the assessment of treatment outcomes and prognostic factors. A review of all available records indicated that these large numbers of cases were representative of the full cohort. However, theoretically, the missing data may have disproportionately affected the results. Therefore, interpretation of these findings is restricted by the limitations of the study design. Retrospectively, asymptomatic patients may have been less likely to attend follow-up, which may have led to an under-reporting of long-term success. A more rigorous study would be prospective and utilize CBCT with software to precisely digitize three-dimensional healing of periapical lesions [41]. Safi et al. showed that success differed by about 8% when evaluated by CBCT versus periapical radiographs [42].

Despite these limitations, the current study involved long-term (over 5 years) follow-ups of a large number of surgical cases that had been performed by the same surgeon (endodontist) using a consistent protocol. This contrasts with other studies that involved multiple operators and surgical techniques, which may confound the analyses of success rates and survival times.

Furthermore, these improvements in success and survival rates obtained through advanced microsurgical techniques are likely to continue with progressive developments in technology. These advancements may include computerized techniques for surgical access with customized trephine burs [43] and dynamic navigation systems using computer-driven optical positioning devices [44]. Future studies will need to investigate treatment outcomes of these next-generation surgical techniques.

## 5. Conclusions

This retrospective cohort study had an overall long-term success rate of 80.5% following endodontic microsurgery. Tooth position, crown restorations, periodontal disease, and maxillary sinus involvement affected the prognosis. Anterior teeth and the presence of crown restorations at follow-up were associated with higher long-term success. Maxillary sinus involvement of periapical lesions and combined periodontal-endodontic lesions were associated with lower success. Additionally, Kaplan–Meier survival analyses showed a 5-year survival rate of 83.5%. Tooth position, crown restoration at follow-up, pain on percussion, RPD abutments, and maxillary sinus involvement had significant effects on survival. Collective evidence supports endodontic microsurgery as a successful and reliable treatment option for treating apical pathosis and maintaining natural teeth.

## Figures and Tables

**Table 1:** Healing outcomes, success, and survival rates.

Outcome	*n*	Proportion (%)
Complete healing	148	65.8
Incomplete healing	33	14.7
Uncertain healing	7	3.1
Unsatisfactory healing ^a^	37	16.4
Success	181	80.5
5-year survival rate ^b^		83.5
10-year survival rate ^b^		83.0

^a^ Teeth extracted within the 5-year follow-up period were included as this category; ^b^ survival rate calculations were based on Kaplan–Meier survival analysis.

**Table 2:** Success rates analyzed (chi-square or Fisher’s exact test) for potential prognostic factors.

Variable		Success		Failure		Chi-Square	*p* Value ^a^
		*n*	(%)	*n*	(%)		
**Age**						4.181	0.041 *
	>50	80	74.8	27	25.2		
	≤50	101	85.6	17	14.4		
Sex						1.898	0.168
	Male	47	74.6	16	25.4		
	Female	134	82.7	28	17.3		
Jaw						0.922	0.337
	Maxilla	140	81.9	31	18.1		
	Mandible	41	75.9	13	24.1		
Tooth position						7.51	0.023 *
	Anterior	82	84.5	15	15.5		
	Premolar	61	84.7	11	15.3		
	Molar	38	67.9	18	32.1		
Hypertension						3.373	0.066
	Hypertensive	28	70.0	12	30.0		
	Normotensive	153	82.7	32	17.3		
Diabetes						Fisher’s exact	0.383
	Diabetes	6	66.7	3	33.3		
	Normal	175	81.0	41	19.0		
Osteoporosis						Fisher’s exact	0.481
	Osteoporosis	2	66.7	1	33.3		
	Normal	179	80.6	43	19.4		
Length of canal filling						1.374	0.503
	Overfilling	11	91.7	1	8.3		
	Underfilling	64	78.0	18	22.0		
	Normal	98	81.7	22	18.3		
Canal filling density						0.46	0.498
	Void	69	83.1	14	16.9		
	No void	104	79.4	27	20.6		
Periodontal disease						Fisher’s exact	0.002 **
	Involvement	3	33.3	6	66.7		
	Not-involved	178	82.4	38	17.6		
Pain						0.239	0.625
	Pain (+)	79	79.0	21	21.0		
	Pain (–)	102	81.6	23	18.4		
Percussion						1.694	0.193
	Present	71	76.3	22	23.7		
	Absent	110	83.3	22	16.7		
Mobility						5.717	0.017 *
	Present	23	65.7	12	34.3		
	Absent	158	83.2	32	16.8		
Palpation						1.19	0.275
	Present	33	86.8	5	13.2		
	Absent	148	79.1	39	20.9		
Bite						0.232	0.63
	Present	20	76.9	6	23.1		
	Absent	161	80.9	38	19.1		
Swelling						<0.001	0.985
	Present	29	80.6	7	19.4		
	Absent	152	80.4	37	19.6		
Sinus tract						1.808	0.179
	Present	33	73.3	12	26.7		
	Absent	148	82.2	32	17.8		
Root resorption						Fisher’s exact	1
	Present	15	83.3	3	16.7		
	Absent	166	80.2	41	19.8		
Crown restoration at follow-up						13.824	< 0.001 **
	Crown	158	84.9	28	15.1		
	None	23	59.0	16	41.0		
Bridge abutment tooth						0.001	0.976
	Yes	25	80.6	6	19.4		
	None	156	80.4	38	19.6		
Tooth opposing implant						Fisher’s exact	1
	Opposing implant	3	100.0	0	0.0		
	None	178	80.2	44	19.8		
RPD abutment						Fisher’s exact	0.007 **
	Yes	0	0.0	3	100.0		
	None	181	81.5	41	18.5		
Post						1.316	0.251
	Present	48	85.7	8	14.3		
	Absent	133	78.7	36	21.3		
Sinus involvement						Fisher’s exact	0.029 *
	Yes	3	42.9	4	57.1		
	None	178	81.7	40	18.3		
Lesion size						1.391	0.238
	>5 × 5 mm	24	88.9	3	11.1		
	≤5 × 5 mm	157	79.3	41	20.7		
Bone graft						Fisher’s exact	0.077
	Bio-Oss	13	100.0	0	0.0		
	None	168	79.2	44	20.8		
Membrane						Fisher’s exact	0.211
	Collagen	9	100.0	0	0.0		
	None	172	79.6	44	20.4		
Re-surgery						Fisher’s exact	0.707
	Re-surgery	9	75.0	3	25.0		
	First surgery	172	80.8	41	19.2		

RPD, removable partial denture; ^a^ *p* value for Chi-square test or Fisher’s exact test. * *p* value < 0.05, ** *p* value < 0.01.

**Table 3:** Results of logistic regression model.

Variables	Beta	SE	*p* Value ^a^	OR	95% Confidence Interval	
					Lower Limit	Upper Limit
Tooth position						
Anterior vs. Molar	1.687	0.601	0.005 **	5.405	1.663	17.571
Anterior vs. Premolar	0.843	0.505	0.095	2.324	0.864	6.249
Age group						
≤50 vs. >50	−0.460	0.396	0.245	0.631	0.290	1.372
Periodontal disease						
Involvement vs. None	−1.772	0.850	0.037 *	0.170	0.032	0.900
Crown at follow-up						
Crown vs. None	2.325	0.566	0.000 **	10.232	3.374	31.024
Maxillary sinus involvement						
Involvement vs. None	−1.675	0.852	0.049 *	0.187	0.035	0.994

SE, standard error; OR, odds ratio; ^a^ *p* value for logistic regression. * *p* value < 0.05, ** *p* value < 0.01.

## Supplementary Materials

The following are available online at https://www.mdpi.com/2077-0383/9/7/2210/s1, Figure S1: Kaplan-Meier survival analysis of teeth following endodontic microsurgery (*n* = 225), according to potential prognostic factors; (a) Tooth position (Anterior vs. Premolar vs. Molar; (b) Crown restoration at follow-up (Overall teeth, Full veneered vs. Non-full veneered); (c) Crown restoration at follow-up (Anterior teeth, Full veneered vs. Non-full veneered); (d) Crown restoration at follow-up (Premolar teeth, Full veneered vs. Non-full veneered); (e) Crown restoration at follow-up (Molar teeth, Full veneered vs. Non-full veneered); (f) Sinus involvement (Involved or not); (g) Percussion (pain or not); (h) RPD abutment (abutment or none). Table S1: Study sample, Table S2: Reasons for extraction, Table S3: Results of the univariate analysis of survival by log rank (Mantel–Cox) test, Table S4: Results of the multivariate Cox proportional hazard regression model.
