# Supplementary material for: Prognostic Factors of Long-Term Outcomes in Endodontic Microsurgery: A Retrospective Cohort Study over Five Years
Source: J Clin Med. 2020 Jul 13;9(7):2210. doi: 10.3390/jcm9072210 (PMC7409012; doi:10.3390/jcm9072210)
Supplement: Supplementary file 1 [file jcm-09-02210-s001.docx]

Supplementary material

Prognostic Factors of Long-term Outcomes in Endodontic Microsurgery: A Retrospective Cohort Study over 5 Years

Yeon-Jee Yoo ^1,†^, Dong-Won Kim ^2,†^, Hiran Perinpanayagam ^3^, Seung-Ho Baek ^2^, Qiang Zhu ^4^, Kamran Safavi ^4^ and Kee-Yeon Kum ^2,5,^*

^1^ Department of Comprehensive Treatment Center, Seoul National University Dental Hospital, Seoul 03080, Korea; duswl32@snu.ac.kr

^2^ Department of Conservative Dentistry, Dental Research Institute, Seoul National University Dental Hospital, Seoul National University School of Dentistry, Seoul 03080, Korea; tokimdw@naver.com (D.W.K.) shbaek@snu.ac.kr (S.-H.B.)

^3^ Schulich School of Medicine & Dentistry, University of Western Ontario, London, ON N6A 5C1, Canada; hperinpa@uwo.ca

^4^ Division of Endodontology, Department of Oral Health and Diagnostic Sciences, University of Connecticut Health Center, School of Dental Medicine, Farmington, CT 06030, USA; qzhu@uchc.edu (Q.Z.); safavi@uchc.edu’(K.S.)

^5^ National Dental Care Center for Persons with Special Needs, Seoul National University Dental Hospital for Persons with Special Needs, Seoul 03080, Korea

***** Correspondence: kum6139@snu.ac.kr; Tel.: +82-2-2072-2656

† These authors contributed equally to this work

Received: 9 June 2020; Accepted: 10 July 2020; Published: date

**Figure S1. Kaplan-Meier survival analysis of teeth following endodontic microsurgery (*n* = 225), according to potential prognostic factors.**

The number of teeth at risk are presented below the x-axis. *P* values are based on the log-rank test. Time 0 on the x-axis (i.e., beginning of teeth being at risk) is date of endodontic microsurgery.

| Anterior vs  Premolar  *P*  value: 0.253  Anterior vs  Molar  *P*  value: 0.019  Premolar  vs  Molar  *P*  value:  0.230  No. at risk  Anterior tooth  97  94  77  49  28  14  3.5  0  Premolar  72  67  52  30.5  13.5  5  1  0.5  Molar  56  51  36.5  23  10.5  1.5  0  0  **A.** **Tooth position (Anterior vs Premolar vs. Molar):** There were 12 extractions in 97 anterior teeth; there were 13 extractions in 72 premolar teeth; there were 15 extractions in 56 molar teeth. Mean survival time was 139.0 months (131.0–149.7 months) for anterior teeth, 138.0 months (122.6–153.4 months) for premolar teeth, and 111.6 months(98.3–124.9 months) for molar teeth. |
| --- |

| No. at risk  Non  -  full veneered  39  36  26  15  6  2  0  0  Full veneered  crown  186  176  139.5  87.5  46  18.5  4.5  0.5  Full veneered vs Non  -  full veneered  *P*  value: 0.005  **B.** **Crown restoration at follow-up (Overall teeth, Full veneered vs Non-full veneered):** Overall survival analysis regarding to restoration status at follow-up. There were 27 extractions in 186 full veneered teeth; there were 13 extractions in 39 non-full veneered teeth. Mean survival time was 147.3 months (140.1–154.6 months) for full veneered teeth, 98.5 months (83.6–113.3 months) for non-full veneered teeth. |
| --- |

| Full veneered vs Non  -  full veneered  *P*  value =  0.012  No. at risk  Non  -  full veneered  33  32  24.5  14  5  1  0  0  Full veneered  crown  64  62  52.5  35  23  13  3.5  0  **C.** **Crown restoration at follow-up (Anterior teeth, Full veneered vs Non-full veneered):** Survival analysis of anterior teeth regarding to restoration status at follow-up. There were 4 extractions in 64 full veneered teeth; there were 8 extractions in 33 non-full veneered teeth. Mean survival time was 145.9 months (138.0– 153.7 months) for full veneered teeth, 109.2 months (95.3–123.1 months) for non-full veneered teeth. |
| --- |
| Full veneered vs Non  -  full veneered  *P*  value  < 0.001  No. at risk  Non  -  full veneered  4  2  1  1  1  1  0  0  Full veneered  crown  68  65  51  29.5  12.5  4  1  0.5  **D.** **Crown restoration at follow-up (Premolar teeth, Full veneered vs Non-full veneered):** Survival analysis of premolar teeth regarding to restoration status at follow-up. There were 9 extractions in 68 full veneered teeth; there were 4 extractions in 4 non-full veneered teeth. Mean survival time was 148.0 months (135.6–160.3 months) for full veneered teeth, 42.8 months (0.0–94.3 months) for non-full veneered teeth. |
| No. at risk  Non  -  full veneered  2  2  0.5  0  0  0  0  0  Full veneered  crown  54  49  36  23  10.5  1.5  0  0  Full veneered vs Non  -  full veneered  *P*  value  = 0.417  **E.** **Crown restoration at follow-up (Molar teeth, Full veneered vs Non-full veneered):** Survival analysis of molar teeth regarding to restoration status at follow-up. There were 14 extractions in 54 full veneered teeth; there were 1 extraction in 2 non-full veneered teeth. Mean survival time was 112.6 months (99.2–126.0 months) for full veneered teeth, 48.5 months (20.1–76.9 months) for non-full veneered teeth. |

| No. at risk  Not Involved  218  207  163.5  102  52  20.5  4.5  0.5  Sinus Involved  7  5  2  0.5  0  0  0  0  Sinus involved vs Not involved  *P*  value = 0.001  **F.** **Sinus involvement (Involved or not):** There were 4 extractions in 7 sinus involved teeth; there were 36 extractions in 218 non-involved teeth. Mean survival time was 48.1 months (25.1–71.2 months) for sinus involved teeth, 144.2 months (137.0–151.5 months) for non-involved teeth. |
| --- |

| Pain to percussion vs Not  *P*  value: 0.022  No. at risk  Percussion (  -  )  132  127  100  60.5  31.5  14  4  0.5  Percussion (+)  93  85  65.5  42  20.5  6.5  0.5  0  **G.** **Percussion (pain or not):** There were 17 extractions in 132 percussion (-); there were 23 extractions in 93 percussion (+). Mean survival time was 149.7 months (141.5–157.9 months) for percussion (-), 114.9 months (104.5–125.3 months) for percussion (+). |
| --- |

| RPD abutment vs None  *P*  value: 0.007  No. at risk  None  222  209  164.5  101.5  51.5  20.5  4.5  0.5  Abutment  3  3  1  1  0.5  0  0  0  **H.** **RPD abutment (abutment or none):** There were 2 extractions in 3 RPD abutment; there were 38 extractions in 222 non-abutments. Mean survival time was 54.0 months (10.8–97.2 months) for RPD abutment, 143.3 months (136.0–150.6 months) for non-abutment.  Abbreviation: RPD, removable partial denture. |
| --- |

**Table S1. Study sample**

| Descriptor | Number | Proportion (%) |
| --- | --- | --- |
| Age (years) |  |  |
| ≤ 50 | 118 | 52.4 |
| > 50 | 107 | 47.6 |
| Sex |  |  |
| Male | 63 | 28.0 |
| Female | 162 | 72.0 |
| Jaw |  |  |
| Maxilla | 171 | 76.0 |
| Mandible | 54 | 24.0 |
| Tooth position |  |  |
| Maxillary Anterior | 82 | 36.4 |
| Mandibular Anterior | 15 | 6.7 |
| Maxillary Premolar | 59 | 26.2 |
| Mandibular Premolar | 13 | 5.8 |
| Maxillary Molar | 31 | 13.3 |
| Mandibular Molar | 25 | 11.6 |

**Table S2. Reasons for extraction**

| Reason | Number | Proportion (%) |
| --- | --- | --- |
| Crown fracture from caries | 7 | 19.4 |
| Root fracture | 5 | 13.9 |
| Periodontal problem | 7 | 19.4 |
| Failure to heal | 6 | 16.7 |
| Persistent pain | 7 | 19.4 |
| Unknown | 4 | 11.1 |
| Total extractions | 36 | 100 |

**Table S3. Results of the univariate analysis of survival by log rank (Mantel-Cox) test**

|  | **Variable** | **Event (*n*) /total** | **Chi-square** | ***P* value ^a^** |
| --- | --- | --- | --- | --- |
| Age |  |  | 4.452 | 0.035 |
|  | > 50 | 25/107 |  |  |
|  | ≤ 50 | 15/118 |  |  |
| Sex |  |  | 0.003 | 0.955 |
|  | Male | 11/63 |  |  |
|  | Female | 29/162 |  |  |
| Jaw |  |  | 0.235 | 0.628 |
|  | Maxilla | 29/171 |  |  |
|  | Mandible | 11/54 |  |  |
| Tooth position |  |  | 5.685 | 0.058 |
|  | Anterior | 12/97 |  |  |
|  | Premolar | 13/72 |  |  |
|  | Molar | 15/56 |  |  |
| Hypertension |  |  | 2.184 | 0.139 |
|  | Hypertensive | 10/40 |  |  |
|  | Normotensive | 30/185 |  |  |
| Diabetes |  |  | 0.117 | 0.732 |
|  | Diabetes (+) | 2/9 |  |  |
|  | Diabetes (-) | 38/216 |  |  |
| Osteoporosis |  |  | 0.724 | 0.395 |
|  | Osteoporosis | 1/3 |  |  |
|  | Normal | 39/222 |  |  |
| Length of canal filling |  |  | 2.965 | 0.227 |
|  | Overfilled | 1/12 |  |  |
|  | Underfilled | 19/82 |  |  |
|  | Adequate | 18/120 |  |  |
| Canal filling density |  |  | 1.719 | 0.190 |
|  | Voids | 11/83 |  |  |
|  | No voids | 27/131 |  |  |
| Periodontal disease |  |  | 7.019 | 0.008 ** |
|  | Involvement | 4/9 |  |  |
|  | Normal | 36/216 |  |  |
| Pain |  |  | 0.235 | 0.627 |
|  | Present | 19/100 |  |  |
|  | Absent | 21/125 |  |  |

|  | **Variable** | **Event (*n*) /total** | **Chi-square** | ***P* value ^a^** |
| --- | --- | --- | --- | --- |
| Percussion |  |  | 5.221 | 0.022 * |
|  | Present | 23/93 |  |  |
|  | Absent | 17/117 |  |  |
| Mobility |  |  | 5.357 | 0.021 * |
|  | Present | 11/35 |  |  |
|  | Absent | 29/190 |  |  |
| Palpation |  |  | 0.508 | 0.476 |
|  | Present | 5/38 |  |  |
|  | Absent | 35/187 |  |  |
| Bite |  |  | 0.811 | 0.368 |
|  | Present | 6/26 |  |  |
|  | Absent | 34/199 |  |  |
| Swelling |  |  | 0.021 | 0.885 |
|  | Present | 6/36 |  |  |
|  | Absent | 34/189 |  |  |
| Sinus tract |  |  | < 0.001 | 0.992 |
|  | Present | 8/45 |  |  |
|  | Absent | 32/180 |  |  |
| Root resorption |  |  | 1.851 | 0.174 |
|  | Present | 1/18 |  |  |
|  | Absent | 39/207 |  |  |
| Restoration at follow-up |  |  | 7.754 | 0.005 ** |
|  | Crown | 27/186 |  |  |
|  | Other | 13/39 |  |  |
| Bridge abutment |  |  | 0.045 | 0.832 |
|  | Bridge abutment | 6/31 |  |  |
|  | None | 34/194 |  |  |
| Tooth opposing implant |  |  | 0.567 | 0.452 |
|  | Opposing implant | 0/3 |  |  |
|  | No | 40/222 |  |  |
| RPD abutment |  |  | 7.346 | 0.007 ** |
|  | Yes | 2/3 |  |  |
|  | No | 38/222 |  |  |
| Post |  |  | 0.002 | 0.965 |
|  | Present | 10/56 |  |  |
|  | Absent | 30/169 |  |  |

|  | **Variable** | **Event (*n*) /total** | **Chi-square** | ***P* value ^a^** |
| --- | --- | --- | --- | --- |
| Anatomic involvement |  |  | 11.888 | 0.001 ** |
|  | Maxillary Sinus | 4/7 |  |  |
|  | Normal | 36/218 |  |  |
| Lesion size |  |  | 1.988 | 0.159 |
|  | > 5 x 5 mm | 2/27 |  |  |
|  | ≤ 5 x 5 mm | 38/198 |  |  |
| Bone graft |  |  | 2.417 | 0.120 |
|  | Bio-Oss | 0/13 |  |  |
|  | None | 36/212 |  |  |
| Membrane | ` |  | 2.559 | 0.110 |
|  | Collagen Membrane | 0/9 |  |  |
|  | None | 40/216 |  |  |
| Re-surgery |  |  | 0.590 | 0.442 |
|  | Re-surgery | 3/12 |  |  |
|  | First surgery | 37/213 |  |  |

Abbreviations: RPD, removable partial denture.
^a^ *P* value for log rank (Mantel-Cox) test.

**Table S4. Results of the multivariate Cox proportional hazard regression model**

|  | Beta | SE | *P* value ^a^ | HR ^b^ | 95% Confidence Interval | |
| --- | --- | --- | --- | --- | --- | --- |
| Variables |  |  |  |  | Lower Limit | Upper Limit |
| Tooth position |  |  |  |  |  |  |
| Anterior vs. Molar | -1.372 | 0.483 | 0.005 ** | 0.254 | 0.098 | 0.654 |
| Anterior vs. Premolar | -0.453 | 0.386 | 0.241 | 0.636 | 0.298 | 1.355 |
| Percussion | 0.732 | 0.341 | 0.032 * | 2.078 | 1.064 | 4.058 |
| Crown at follow-up | -1.796 | 0.418 | 0.000 ** | 0.166 | 0.073 | 0.376 |
| Anatomic involvement | 1.399 | 0.550 | 0.011 * | 4.049 | 1.378 | 11.900 |
| Removable partial denture abutment | 2.176 | 0.779 | 0.005 ** | 8.813 | 1.914 | 40.576 |

Abbreviations: SE, standard error; HR, hazard ratio; RPD, removable partial denture

^a^ *P* value for multivariable-adjusted Cox proportional hazards regression.

^b^ Cox proportional hazards regression model adjusted for tooth position, percussion, crown at follow-up, sinus involvement, and RPD abutment. Age, periodontal disease and mobility were excluded by backward elimination using likelihood ratio.
